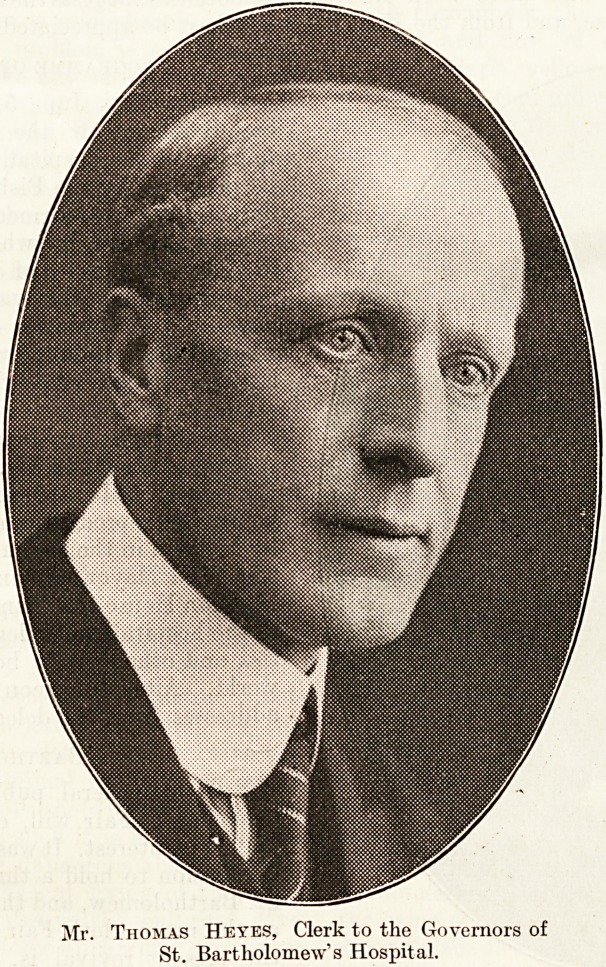# St. Bartholomew's Octocentenary

**Published:** 1923-06

**Authors:** 


### ST. BARTHOLOMEW'S OCTOCENTENARY.

### FAIR AND PAGEANT.

THE octocentenary celebrations in connection with St. Bartholomew's Hospital begin on Tuesday, June 5, and will continue throughout the ensuing week. From the hospital's point of view the principal events in the celebrations will be the publication of a Commemorative Volume with an outline of its history and its needs, edited by Sir D'Arcy Power; the preparation of architectural plans for its gradual reconstruction, by Mr. T. A. Lodge, A.R.I.B.A.; and the presentation of a commemorative medal, designed by Mr. C. L. Hartwell, A.R.A., to the delegates and others who have been invited o attend from every diocese, and from the English-speaking universities and other learned and professional bodies. The outline of the hospital's history should have been a relatively easy matter, since Sir Norman Moore published his monumental volumes four years ago. They proved the author's contention that ever since its foundation the hospital's story has been interwoven with that of London and England. The institution was a healthy, vigorous city growth, as the revival, on this anniversary, of St. Bartholomew's Fair should remind us. It was St. Bartholomew who, in a vision, encouraged Rahere to found the institution. Henry I. granted him the site he desired, and the first charter was given in 1133. The first miracle performed at the Church was an orthopædic one, and enabled a cripple to walk. Rahere died in 1145, but the hospital's independence was preserved by a series of charters that prevented it after the Reformation from sharing the Priory's fate. In 1212, the hospital began the relations with the Mayors of London which the revival of the Fair recalls to-day. These dips into history could be multiplied a hundred times, but their very remoteness suggests the mood in which the celebrations can best be appreciated.

*Photo: Swaine.*]
Miss A. McIntosh, C.B.E., R.R.C., Matron, St. Bartholomew's Hospital.

### Programme of the Celebrations.

On Tuesday, June 5, a service will be held at St. Bartholomew the Great, where Rahere, the founder of the hospital, is buried, and a sermon will be preached by the Bishop of Chester, the son of Sir James Paget, a former Bart.'s man. The service will be followed by what is called a "Solemnity" in the quadrangle of the hospital, attended by delegates from the various universities and the governors and friends of the institution, when the Augustinian canons now in England will sing their ancient hymn. A brief representation of two episodes in the history of Bart.'s will be given. These episodes are being arranged by Mr. Richard Atkins, of the "Old Vic." Theatre, while Mr. Arthur Bourchier will represent Henry VIII., his dress being a reproduction of that in the portrait by Holbein in the committee-room of the institution. The Lord Mayor will entertain to luncheon at the Mansion House the Prince of Wales, who is president of the hospital, and delegates from hospitals, universities and other public bodies in many parts of the world. After luncheon the Prince will receive addresses from the delegates at the Guildhall.

### St. Bartholomew's Fair.

For the general public the revival of St. Bartholomew's Fair will, doubtless, be the principal matter of interest. It was Rahere himself who received permission to hold a three-days' fair on the feast of St. Bartholomew, and this became a great cloth mart, as the name Cloth Fair reminds us. The model for the present revival is, however, the fair of Elizabethan times, when wholly secular rejoicings were the rule and the fair lasted for a fortnight. Then, as will be the case this year, the Lord Mayor opened the festivities. Ben Jonson's play, recently revived, gives a literary picture of them. Already in the seventeenth century the fair began to have critics, and when the Lord Mayor went to it in 1850, its character had become such that neither he nor his successors proclaimed its opening any more. Owing to limitations of space, not more than 20,000 tickets (5s. the first day, 2s. 6d. the second) will be issued in all, and must be purchased in advance. On the Wednesday and Thursday, tableaux arranged by Sir Aston Webb, President of the Royal Academy, will be given in the Great Hall, illustrative of the history of the hospital. On Wednesday there will be a private reception of the delegates at the Royal College of Surgeons and a dinner of old students of the hospital.

OTHER ARRANGEMENTS.

On the Thursday, besides the fair and tableaux, there is to be an evening conversazione in the hospital and medical college; on the Friday a meeting of the Rahere Masonic Lodge; and on the Saturday there will be a cricket match, Past *v.* Present, at the hospital sports grounds, Winchmore Hill, and the League of St. Bartholomew's Nurses will be "at home" to the delegates in the Great Hall. On Sunday, June 10, there will be services at the Priory Church of St. Bartholomew's the Great at 11 a.m. and 6.30 p.m., when seats will be reserved for delegates.

THE "BART'S." OF TO-DAY.

But, after all, the long history of the hospital, and the secular festivities associated with it, are merely reminders of the tradition it has to maintain, and of the developments that still await it. It is interesting, therefore, that the octocentenary will see the completion of the first block of the new Nurses' Home, Queen Mary's Home for St. Bartholomew's Nurses. The block is named after the late Constance Lady Stern, and the Home owes much to Sir Edward Stern's generosity. An account of the hospital's needs and prospective developments is contained in the Commemorative Volume alluded to above, and here we can only touch upon some of them.

NEW ACTIVITIES.

Recent improvements include a Samaritan Fund for out-patients, to do for them what the similar fund has accomplished for in-patients, and the reorganisation of the out-patients' inquiry department, to which an "out-patients' interviewer" has been appointed. A "Follow-up" department has also been provided on the advice of the medical staff to ascertain the ultimate value of the treatment given in the wards, especially in regard to cases suffering from diabetes mellitus and carcinoma. The amalgamation with the Queen Alexandra Hospital for Children with hip disease was accomplished last November, and the work has been made the subject of an independent report. The Dunn Laboratories, provided by the grant of £10,000 from the late Sir William Dunn's trustees, are being formed by adopting the old isolation block, and will be connected by a bridge with the professorial wards on the first floor of the south wing. The isolation bungalow, thereby necessitated, is already occupied, and its cost is being met out of the above-named grant.

MR. THOMAS HEYES, Clerk to the Governors of St. Bartholomew's Hospital.

THE NEAR FUTURE.

An operation block is in contemplation, but Mr. Lodge's scheme, favoured by the Standing Committee of Surgeons, would involve the reconstruction of the south wing, which at present the hospital's finances hardly permit. A Preliminary Training Home for Probationers, the need for which has long been urged, is likely to come forward shortly. The completion of the first block of Queen Mary's Home for St. Bartholomew's Nurses, however, should make it possible to house in the hospital those nurses hitherto occupying the Probationers' Home in King Square, Goswell Road, and thus allow the latter to be adapted for use as a Preliminary Training Home.

GROWTH AND VITALITY.

The moral of these accomplished or projected changes is that the octocentenary celebrations coincide with a period of vigorous growth, and at no time has the vitality of the institution been greater than it is to-day after eight hundred useful years. A real opportunity is here provided to take stock of the past and the future, and we hope that all who realise the significance of these celebrations will turn to the commemorative volume and learn from Sir D'Arcy Power the extent of the developments awaiting the hospital whose long history is now to be recalled to Londoners and indeed the country at large.

**Finances of St. Bartholomew's.**

In his report for the year 1922 the Treasurer of St. Bartholomew's Hospital announces an excess of income over expenditure of £23,920 19s. 8d.; but against this has been set off a debit balance of £12,117 6s. 11d. from the previous year, an expenditure of £2,041 5s. on account of the Medical School incurred in 1920, the repayment of instalments of mortgage, £1,791 12s. 5d., and donations and legacies received in the form of stock amounting to £5,348 9s. 9d. transferred to capital.

## Figures and Tables

**Figure f1:**
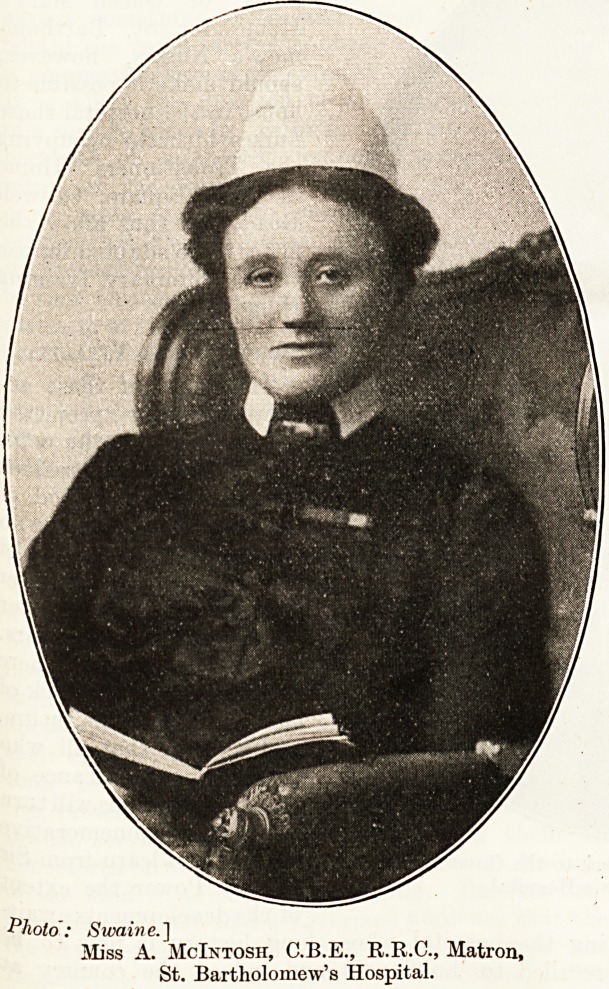


**Figure f2:**